# COVID-19, economic crisis, and food insecurity worsen the double burden of malnutrition in Lebanon

**DOI:** 10.3389/fpubh.2024.1333565

**Published:** 2024-03-05

**Authors:** Alissar Al Khatib

**Affiliations:** Department of Nursing, Almoosa College of Health Sciences, Al Ahsaa, Saudi Arabia

**Keywords:** COVID-19, food insecurity, double burden, malnutrition, Lebanon

## 1 Introduction

Malnutrition is defined as the excesses, imbalances, or deficiencies in a person's dietary intake. In all its forms, malnutrition comprises two large groups: undernutrition (wasting, stunting, underweight, and micronutrient deficiencies) and the second group, which covers overweight, obesity, and their resulting diet-related non-communicable diseases (1). Malnutrition happens in urban and rural areas alike since urbanization, poverty, economic development, and lifestyle behaviors are the most common causes of malnutrition across high- and low-income countries (2). Food insecurity (FI), one of the most important leading causes of malnutrition, is defined as “a situation that exists when people lack regular access to adequate amount of safe and nutritious food for normal growth and development and an active and healthy life” (3). During the past decade, there was a significant global rise in the level of moderate-to-severe food insecure people, which accounts for almost 30% of the world's population, with more than 50% of them hailing from Asia, and 33% from Africa (4).

## 2 The global burden of malnutrition

According to the WHO, the double burden of malnutrition is the coexistence of undernutrition along with overweight/obesity or diet-related non-communicable diseases (5). Worldwide, in 2020, 1.9 billion were overweight, with body mass index (BMI) between 25 and 29.9, or obese (BMI > 30), while 462 million adults were underweight (BMI < 18.5). For children under five, 149 million, 45 million, and 38.9 million were estimated to be stunted, wasted, and overweight, respectively. Moreover, in low- and middle-income countries, undernutrition resulted in ~45% of deaths among children under five, along with an increase in the rates of overweight and obesity in these countries (1). In fact, in the Arab region, the double burden of malnutrition at the individual level, such as stunting coexisting with overweight in children, is twice as high as in other countries worldwide and has exceeded expectations (6). Therefore, the Arab region countries are facing an overlap of overnutrition and undernutrition, mainly in Palestinian and Syrian refugee populations in Lebanon (7, 8).

### 2.1 The health burden of malnutrition in Lebanon

Lebanon is one of the countries in the Arab region that has shown rapid urbanization, changes in its food system, and accelerated transition in nutrition norms despite political challenges and conflicts. However, in 2019, the Lebanese economic crisis affected the official currency as the Lebanese pound collapsed and lost more than 90% of its value (1), diminishing the population's accessibility to basic needs such as food, water, education, and healthcare services. Accordingly, the prices of food increased by 550% between 2020 and 2021 (9). The United Nations (UN) declared that in 2021, ~78% of the Lebanese population lived in poverty, which was three times higher than the estimated number in 2020 (10). On the other hand, the World Bank declared in its latest reports on food security worldwide that Lebanon achieved the highest nominal food price inflation rate in the world during the February 2022–February 2023 period. The country registered a 261% annual change in the food Consumer Price Index (CPI), followed by Zimbabwe (128%) (11). Living below the national poverty line will increase food insecurity, as well as hidden hunger, within the Lebanese population, raising the risk of developing the problem of malnutrition in the coming years.

### 2.2 National nutrition and food insecurity

Since 2019, the socioeconomic situation in Lebanon has worsened further due to the COVID-19 pandemic and the measures implemented to control it. The worsening of the economic status of the Lebanese population led to a reduction in the monthly income and a shortage in food availability and affordability. Therefore, food insecurity increased during these difficult times with the country facing many financial, political, economic, and health crises. Lebanon urgently needs policies to ensure food security and alleviate the burden of the current situation on its population, especially Lebanese children who are at a higher risk of malnutrition (12). Therefore, the majority of the Lebanese population has been plunged into poverty, with 22% of Lebanese being food-insecure due to considerable inequities in access to healthy foods across the population (13). In 2021, Lebanon's Ministry of Public Health conducted a survey supported by the United Nations International Children's Emergency Fund (UNICEF) and Action Against Hunger (AAH); the survey revealed that ~200,000 children under 5 are suffering from at least one form of malnutrition, including wasting, stunting, and micronutrients deficiencies (13). A recent study in Lebanon showed that ~37.6% and 37.4% had moderate and severe food insecurities. In addition, participants who reported a bad overall health status of their children declared that they were severely food insecure. Moreover, those with severe food insecurity stated that their children's daily snacking habit between meals had decreased, with a decreased quantity of meals and intake of vegetables/fruits. Moreover, severe food insecurity was significantly associated with higher mean financial burdens than the other groups (12).

In Lebanon, the prevalence of wasting and underweight is found to be 1.1 and 3.71%, respectively (14), where wasting drivers include inadequate diet and services, poor practices, and disease. Furthermore, more than 70% of children are not receiving breastfeeding, only 6% were consuming minimum acceptable diets, and 54% of survey participants tend to reduce portions of meals when they do not have enough food, rendering 1.8 and 5% of children and pregnant and lactating women malnourished. According to the survey, stunting was recorded at 7%, with a prevalence slightly higher in the capital city than in the other governorates with levels similar to the national stunting prevalence (13).

**Figure 1:**
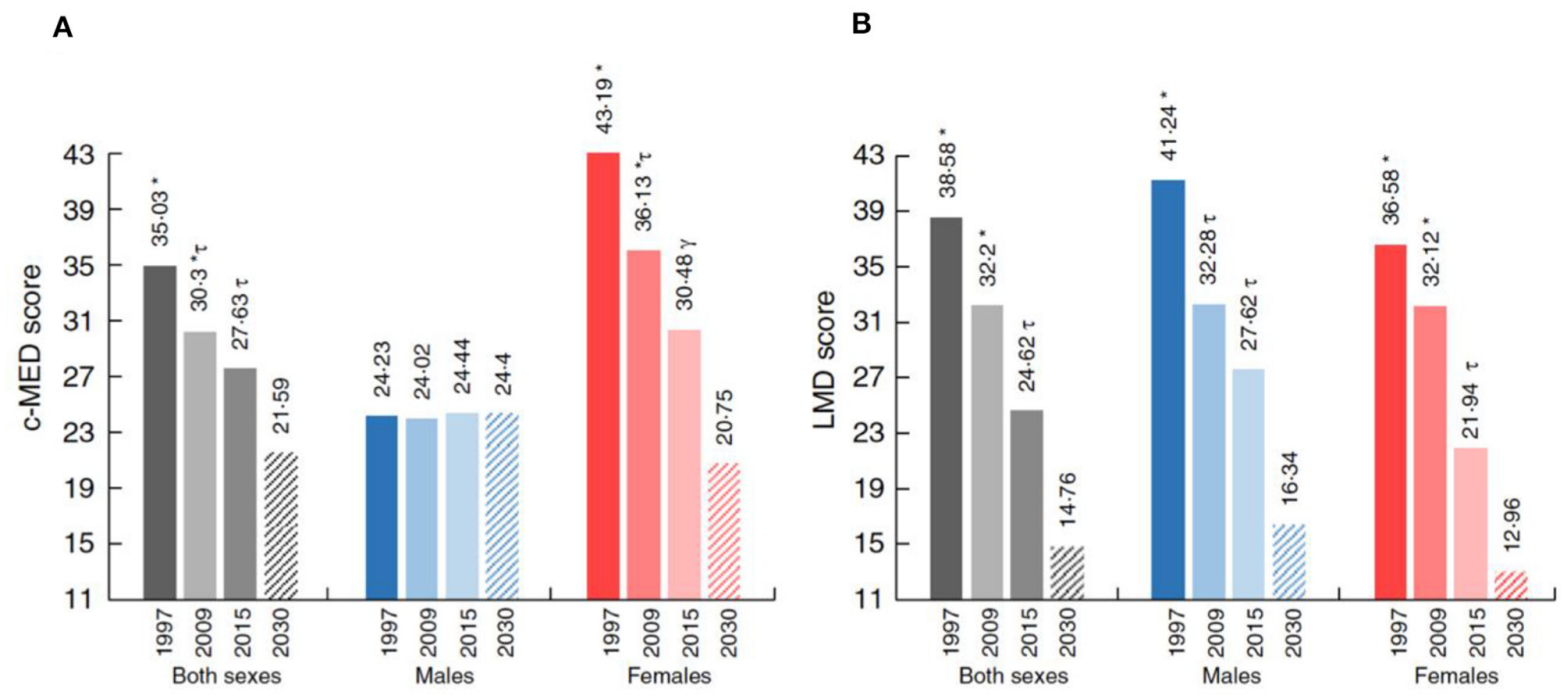
**(A, B)** Proportion of the study population's level of adherence to the Mediterranean diet scores by sex and survey year and projection to 2030. c-MED, composite Mediterranean diet; LMD, Lebanese Mediterranean diet. Projections are presented by the dashed rectangles. *, τ, γDifferent symbols indicate significant difference between the corresponding groups.

On the other hand, adequate dietary intake during childhood is a key component for promoting health and development as well as for disease prevention throughout a lifetime (15). In Lebanon, the last few years have shown an alarming rise in the rates of overweight (21.2%) and obesity (10.9%) among school-age children, higher than the global estimate of 6.7% (16); such an increase could be explained by the rapid transition in lifestyle behavior (less physical activities, more addiction to video games) and due to the switch from the Lebanese Mediterranean Diet rich in fruits, vegetables, grains, olive oil, and protein to a Westernized dietary pattern with high fat, sugar, and salt contents. Several studies have shown that this transition in dietary patterns is correlated with an increase in the rate of overweight and obesity, as well as the increase in the risk of early development of non-communicable diseases among children (17–19). Accordingly, a recent study has estimated that by 2030, only 14%−20% of adolescents in Lebanon will remain adherent to the Mediterranean Diet pattern as a projection from the norm of food consumption trend of Lebanese children between 1995 and 2009 (20) (Figure 1). Several studies provide considerable evidence for the importance of the Mediterranean diet in protection against non-communicable diseases, mainly diabetes, hypertension, cancer, and cardiometabolic risk factors (18). On the other hand, based on the national survey conducted among Lebanese preschoolers, ~9% of Lebanese children under five are classified as overweight or obese (14); this study revealed that overweight and obesity in this age group was significantly associated with the presence of a household maid, which affected the children's adherence to the traditional pattern, in addition to the high intake of sweets and beverages with added sugar.

### 2.3 Double burden of malnutrition

**Figure 2:**
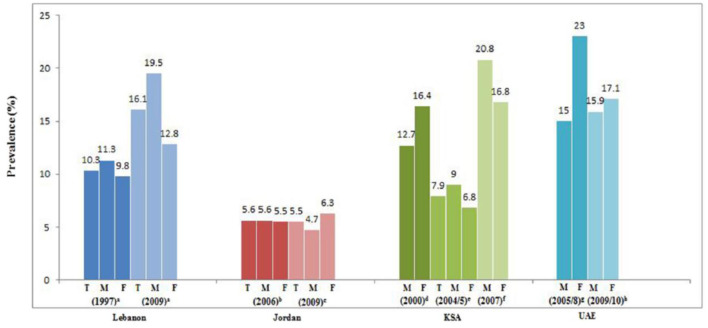
Prevalence (%) and trends of obesity in school-aged children in the eastern Mediterranean region. T, total; M, Males; F, Females (Lebanon: left/blue). Source: Nasreddine et al. (21). a: FAO (22); b: Food and Agriculture Organization (FAO) (23); c: Assaf and Bradley (24); d: Hossain et al. (25); e: Miller et al. (26); f: Ministry of Health (MOH) (27); g: Khatib and Elmadfa (28); h: Ministry of Health (MOH) (29).

**Figure 3:**
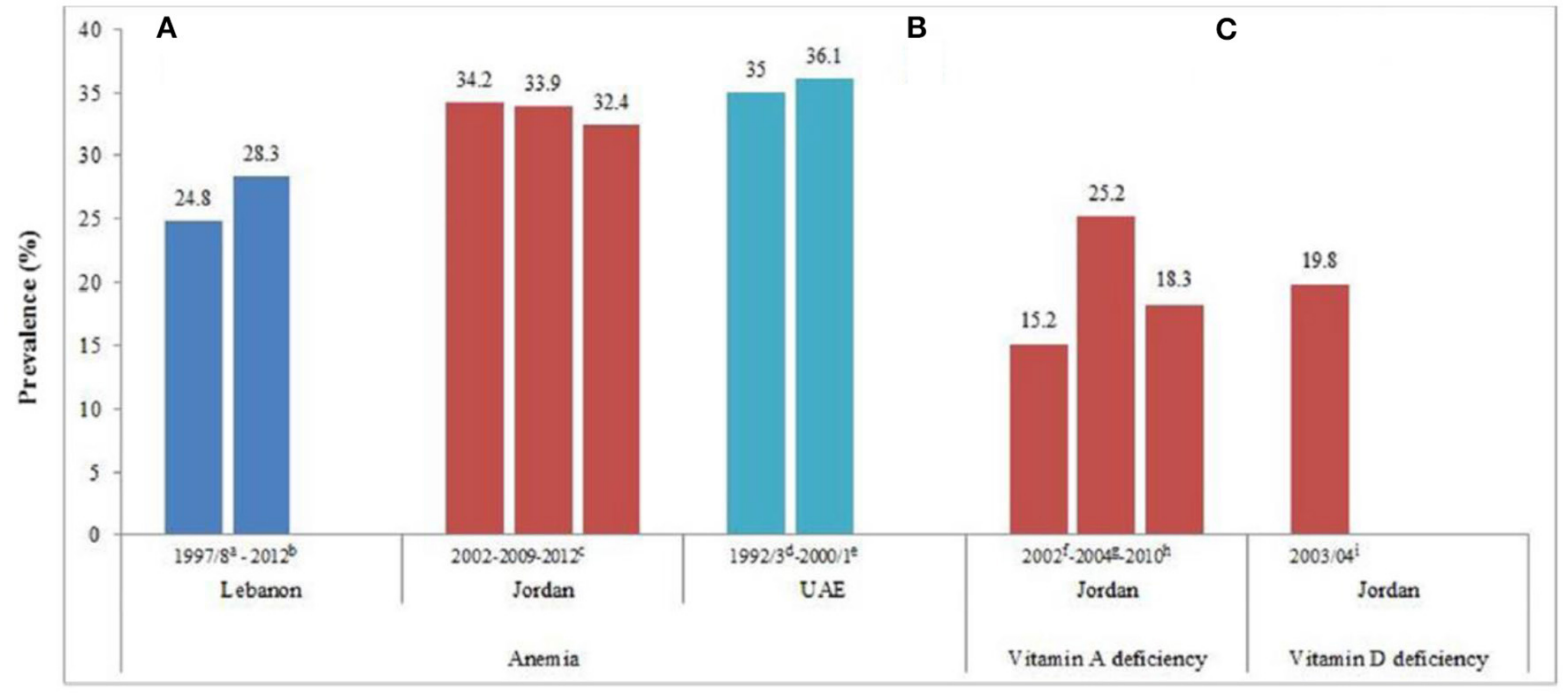
**(A–C)** Prevalence (%) of anemia, vitamin A deficiency, and vitamin D deficiency in preschool children in the Eastern Mediterranean Region. Abbreviation: UAE, United Arab Emirates. a: FAO (22); b: Food and Agriculture Organization (FAO) (23); c: Assaf and Bradley (24); d: Hossain et al. (25); e: Miller et al. (26); f: Ministry of Health (MOH) (27); g: Khatib and Elmadfa (28); h: Ministry of Health (MOH) (29); i: Ministry of Health (MOH) (30).

**Figure 4:**
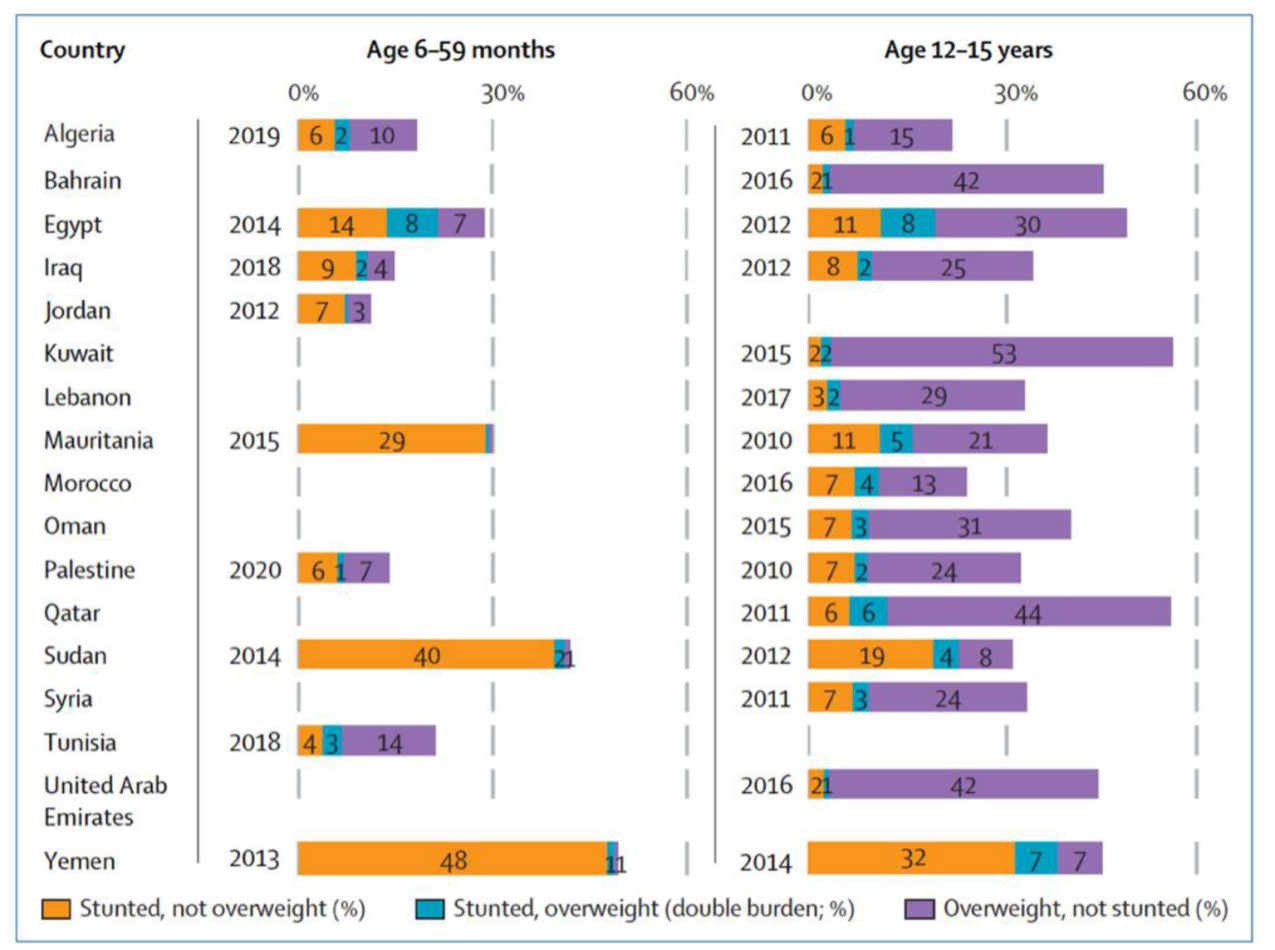
Prevalence of stunting, overweight, and individual-level double burden in adolescents aged 12–15 years in Lebanon (2017), no data available for children aged 6–59 months. Source: Ghattas et al. (8).

Lebanon is classified as a low-income country in early nutrition transition. Studies revealed that Lebanon is one of the countries in the Arab region manifesting an increasing trend in the prevalence of obesity in school-age children along with an anemia prevalence of 28.3% (Figures 2, 3). Moreover, iron, vitamin D, and iodine deficiencies reached 11%, 21%, and 56%, respectively, in 1998 (21, 22, 31). Therefore, if obesity is attributed to bad lifestyle behavior, such as lack of physical activities and consumption of unhealthy foods with high sugar and fat contents, micronutrient deficiency or hidden hunger is another form of malnutrition correlated with poor adherence to the average daily intake level of nutrients. Accordingly, several studies reported an inadequate micronutrient intake among this age group, where 35%, 84%, 85%, and 95% did not meet two-thirds of the Recommended Daily Allowance (RDA) for iron, calcium, copper, and vitamin D, respectively (21, 32). As mentioned previously, Lebanese school-age children showed a high prevalence of obesity coexisting with micronutrient deficiencies, which highlights the double burden of malnutrition in Lebanon. On the other hand, when the prevalence of stunting in Lebanon was compared with the World Health Assembly recommendation for a reduction in the number of children under 5 years who are stunted and wasted by 40% and 5%, respectively, as targets for 2025 (33), Lebanon seems to be on the right track. However, in light of the economic crisis, the rates of malnutrition are expected to rise even more in the upcoming years (13). Therefore, actions should be taken to reduce the double burden of malnutrition in Lebanon, especially the risks of non-communicable diseases, which accounted for 71% of all deaths in 2018 (34). One of the implemented actions is the school feeding programs (SFP) in collaboration with the World Food Program, where fruits, vegetables, nuts, and milk are introduced instead of high-energy snacks to control the double burden of malnutrition among this age group in Lebanon (Figure 4) (8).

### 2.4 Determinants and consequences of malnutrition

Malnutrition is attributed to individual lifestyle behavior, socio-demographic characteristics, and economic, physiological, and pathological factors (35). In Lebanon, Mitri et al. (36) reported that among older adults aged 65 years and above, women were significantly more at risk for malnutrition than men. The risk of poor nutrition increased with low level of education and the absence of health insurance and fixed income. Moreover, the population with comorbidities and taking several medications was significantly at higher risk for malnutrition than those with good health and practicing daily physical activities. Surprisingly, oral health in adults, mainly older individuals, can affect their nutrition and may put them at risk of poor nutrition when dentures cannot cover their nutritional needs (36). Furthermore, depression and BMI are among the socio-demographic variables that are significantly associated with a greater risk of malnutrition among Lebanese older adults (35). For school-age children, malnutrition was mainly associated with age, time spent on electronic devices, skipping meals, and inadequate intake of nutrients (37). Among micronutrient deficiencies that affect the physical growth, psychological development, and academic performance of children, Vitamin A, Vitamin D, and pediatric anemia represent a great challenge in Lebanon. Moreover, studies have shown that the increase in overweight and obesity prevalence in Lebanon among school-age children raises the risk for type 2 diabetes mellitus, hypertension, and cardiovascular diseases among this age group and worsens the burden of non-communicable diseases in the country in the long term (16).

## 3 Data reliability

Amid the political challenges and socioeconomic crisis faced by Lebanon in recent years, the country has hosted a large number of refugees, with an estimated 1.5 million and 14,815 refugees of Syrian and other nationalities, respectively (38). The majority of Syrian refugees do not hold legal residency in Lebanon; thus, they suffer from a lack of healthcare services and have no access to basic daily needs such as healthy food and water. Moreover, the attention of humanitarian organizations was transferred to this population living in extreme poverty. Moreover, most surveys, studies, data collections, and analyses focused on the living conditions of Syrian and Palestinian refugees in their camps and ignored the national sector. Therefore, we have encountered difficulties in describing and analyzing the burden of malnutrition in Lebanon, especially among older Lebanese adults aged 65 years and older. This age group is expected to account for 10.2% of the total population in 2025, according to projections (39). Moreover, Choueiry et al. (35) reported that since 2008, there has been no update on the nutritional status of hospitalized Lebanese patients; the nutritional survey of rural older adults was recently targeted in 2013 (36), and not enough data for vitamin A deficiency among Lebanese population has been published (21).

## 4 Mitigating the burden of malnutrition through health system approach

Vertical and horizontal approaches are used in health systems planning to address health problems (40). Tackling malnutrition across the population suffering from the double burden of malnutrition should include two main levels: First, the implementation of interventions at the individual level through a vertical approach; and second, multi-level actions by collaborating with different governmental sectors through a horizontal approach for policy development and enactment. In addressing undernutrition, schools can help significantly in the prevention and management of micronutrient deficiencies among this age group by providing effective nutrition education. Therefore, the organization of nutrition campaigns in schools is the best way to increase children's knowledge of healthy food choices and to improve their adherence to the recommended dietary intake guidelines. The outcomes of the vertical approach are time-limited, with rapid interactions and responses to the implemented interventions. The effectiveness of the previously mentioned approach is justified by one of the most important implemented nutrition interventions in Lebanon under the title “*Your health is on your plate”* where Said et al. (41) have demonstrated the great outcomes of implemented action in increasing knowledge and adherence to dietary guidelines in Lebanese adolescents. However, policies that fight overnutrition across the population through a horizontal approach need the engagement of several sectors, such as stakeholders, health, justice, environment, agriculture, and education. These sectors interact together for policy development and enactment to control the availability of energy-dense food and sweetened drinks at local stores, enhance physical activities at schools by limiting screen time access, enhance breastfeeding, and raise parental awareness toward a healthy lifestyle. Serious strategies and effective interventions should be implemented to avoid further inflation and ensure food security for the Lebanese population, such as encouraging internal agriculture, decreasing food prices, food-wasting, and unemployment, and improving infrastructural programs, trade policies, and diversification.

The success of implemented approaches could be evaluated through the Health Economic Evaluation and Health Impact Assessment (HIA), which assesses the health and equity impact of the implemented policies. This allows us to not only evaluate the success of interventions but also enables early detection of problem to find solutions. Several studies have shown the significant association between parent's socio-demographic characteristics, education level, maternal BMI, and child feeding practices (19). Therefore, response strategies are mandatory to avoid further hunger and the impact of food insecurity on human physical and mental health.

## 5 Conclusion

Currently, the burden of malnutrition is considered to be one of the most important public health challenges. Malnutrition in all its forms has short- and long-term complications that affect the psychological and physical wellbeing of children and adults. Therefore, a multi-sectoral collaboration between stakeholders, non-governmental organizations, social protection, and welfare and funding agencies is highly needed to address the root causes of malnutrition and decrease the attributed risk factors earlier in life (42).

## Author contributions

AA: Conceptualization, Writing—original draft.
